# Transcriptomic profiling across human serotonin neuron differentiation via the FEV reporter system

**DOI:** 10.1186/s13287-024-03728-x

**Published:** 2024-04-19

**Authors:** Yingqi Li, Jinjin Duan, You Li, Meihui Zhang, Jiaan Wu, Guanhao Wang, Shuanqing Li, Zhangsen Hu, Yi Qu, Yunhe Li, Xiran Hu, Fei Guo, Lining Cao, Jianfeng Lu

**Affiliations:** 1 Shanghai YangZhi Rehabilitation Hospital (Shanghai Sunshine Rehabilitation Center), Frontier Science Center for Stem Cell Research, School of Life Sciences and Technology, Tongji University, Shanghai, China; 2 Institute of Biophysics, Chinese Academy of Sciences, Beijing, China; 3 College of Life Sciences, University of Chinese Academy of Sciences, Beijing, China; 4 Key Laboratory of Receptor Research, Shanghai Institute of Materia Medica, Chinese Academy of Sciences, Shanghai, 201203 China; 5 Suzhou Institute of Tongji University, Suzhou, China

**Keywords:** Serotonin neurons, FEV-EGFP reporter, Human pluripotent stem cells, Differentiation, CRISPR/Cas9

## Abstract

**Background:**

The detailed transcriptomic profiles during human serotonin neuron (SN) differentiation remain elusive. The establishment of a reporter system based on SN terminal selector holds promise to produce highly-purified cells with an early serotonergic fate and help elucidate the molecular events during human SN development process.

**Methods:**

A fifth Ewing variant (FEV)-EGFP reporter system was established by CRISPR/Cas9 technology to indicate SN since postmitotic stage. FACS was performed to purify SN from the heterogeneous cell populations. RNA-sequencing analysis was performed for cells at four key stages of differentiation (pluripotent stem cells, serotonergic neural progenitors, purified postmitotic SN and purifed mature SN) to explore the transcriptomic dynamics during SN differentiation.

**Results:**

We found that human serotonergic fate specification may commence as early as day 21 of differentiation from human pluripotent stem cells. Furthermore, the transcriptional factors ZIC1, HOXA2 and MSX2 were identified as the hub genes responsible for orchestrating serotonergic fate determination.

**Conclusions:**

For the first time, we exposed the developmental transcriptomic profiles of human SN via FEV reporter system, which will further our understanding for the development process of human SN.

**Graphical Abstract:**

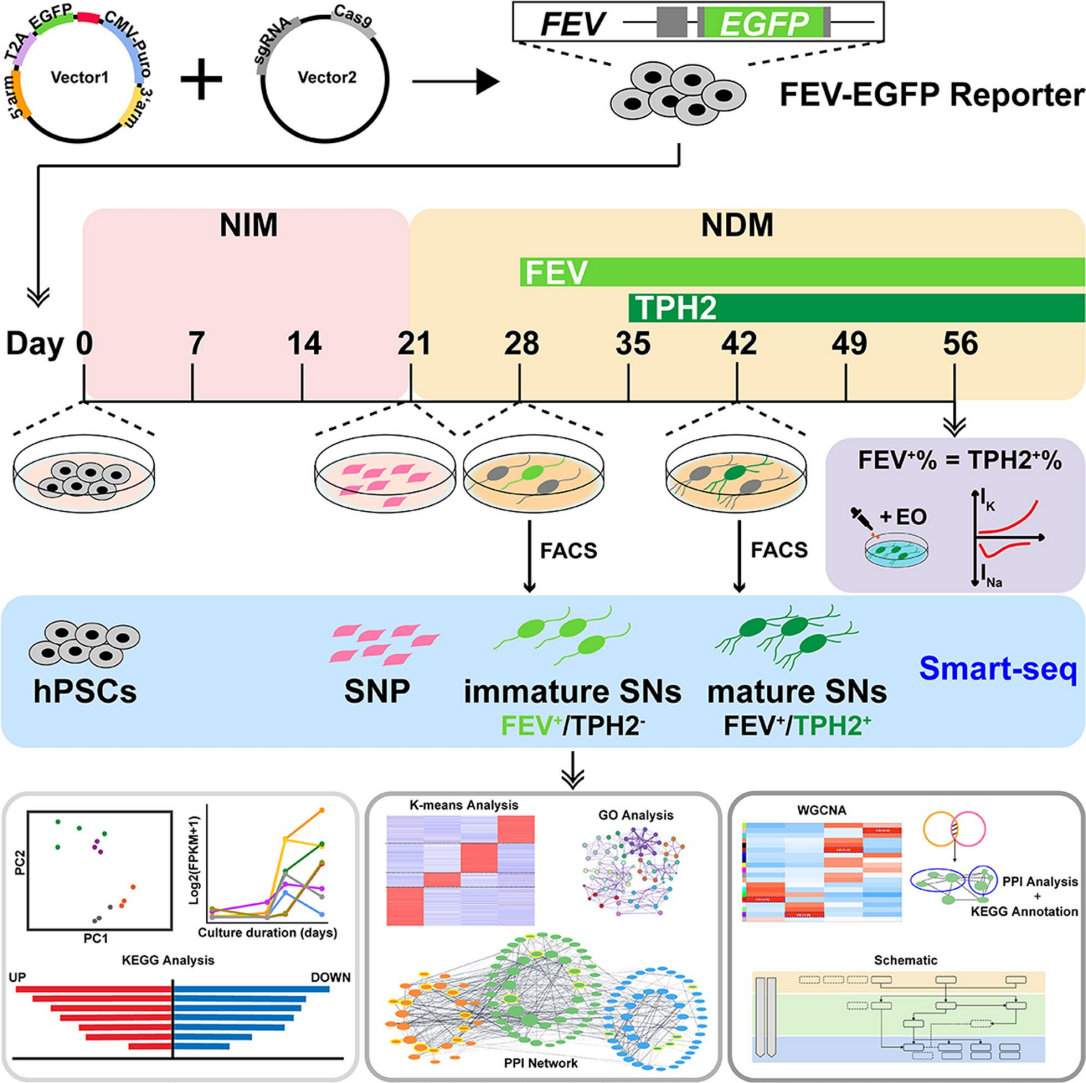

**Supplementary Information:**

The online version contains supplementary material available at 10.1186/s13287-024-03728-x.

## Introduction

More attention has been directed toward the role of serotonin neurons (SN) since dysregulation of serotonergic system has been considered as a crucial factor associated with various neuropsychiatric disorders [1, 2], substance abuse and vulnerability to drug relapse [3]. Human pluripotent stem cells (hPSC)-derived SN provide valuable cellular model for studying the related disorders. In 2016, we successfully established a protocol to differentiate hPSC into SN in vitro, which expressed serotonergic markers, produced and released serotonin, and displayed functional electrophysiological properties [4].

Nevertheless, due to lack of the understanding of the molecular cues involved in the development of human SN, the direct differentiation of hPSC into SN still encounters several challenges. A variety of cell types with different degrees of maturity and fate potentials were inevitably produced during SN differentiation process, which negatively influence the yields of SN, thereby hampering its utility in translational applications, including high-throughput drug screening, disease modeling and personalized medicine research. Previously, we established a TPH2-reporter system by using CRISPR/Cas9-mediated gene-editing technology, which enabled us to label TPH2-positive human SN with EGFP for subsequent analysis, such as fluorescence-activated cell sorting (FACS)-mediated purification, drug screening, electrophysiological detection and RNA-sequencing [5]. This system also helped us to elucidate the role of FOXA2 in human SN development [6]. However, the TPH2-reporter system can only indicate mature SN instead of early-stage immature SN, making it impossible to explore the mechanisms governing early patterning and cell fate specification during human SN differentiation. Therefore, establishing a reporter system indicating early-stage SN may help uncover the whole picture of human SN development.

Accumulating studies have elucidated the transcriptomic mechanisms underlying the development of mouse SN, although most of them primarily focused on understanding the molecular events occurring after the acquisition of serotonergic identity. It is revealed that a series of transcription factors regulated by Sonic hedgehog (SHH) and WNT signaling pathways collaborate to specify the serotonergic fate [4, 7, 8]. Particularly, the mouse ETS-domain transcription factor *Pet1* is identified to be indispensable for the terminal induction of mouse SN [9, 10]. Mouse *Pet1* is exclusively expressed in the central serotonergic system with its onset consistently preceding the emergence of 5-HT by a half day [10]. It plays a dual role of establishing and maintaining the serotonergic phenotype, as well as interacting with the regulatory regions of serotonergic phenotype-associated genes, including serotonin transporter (*5-HTT*), 5-HT1a receptor, tryptophan hydroxylase and aromatic L-amino acid decarboxylase [10]. Mice lacking *Pet1* fail to develop into the majority of central SN, and the remaining SN show deficient expression of genes essential for 5-HT synthesis, uptake and vesicular storage [9]. As the orthologous gene to mouse *Pet1*, the human fifth Ewing variant (*FEV*) gene is exclusively expressed by the raphe SN in human central nervous system [11]. Given its approximately 96% homology to the predicted mouse PET1 protein, the comparable restricted expression pattern in the central serotonergic system and the similar function as PET1 [11–13], it is anticipated that FEV plays a similar role in the development of human SN. Although there have been numerous studies investigating the role of PET1 for mouse SN development, there remains an unresolved knowledge gap regarding the precise timing of FEV expression and its specific function in human SN development, which hinders the demonstration of the transcriptional regulatory mechanisms underlying the development of human SN. Therefore, the establishment of FEV reporter system will help to monitor FEV expression in a real-time manner during human SN differentiation process; moreover, highly purified FEV-positive early-stage human SN will facilitate the understanding of the developmental transcriptomic network of human SN.

In the present study, we successfully constructed an FEV-EGFP reporter system by using CRISPR/Cas9-mediated gene-editing technology. Our investigation revealed that FEV expression is earlier than TPH2 expression. This FEV-EGFP reporter system enabled us to produce immature SN with a high degree of purity, all of which subsequently differentiated into TPH2-positive mature SN. Furthermore, the transcriptional factors *ZIC1*, *HOXA2* and *MSX2* were identified as hub genes responsible for orchestrating serotonergic fate determination. With the FEV reporter system, we exposed the developmental transcriptomic profiles of human SN for the first time, which will help to understand the crucial molecular events during human SN development and to facilitate the application of human SN in disease modeling, drug screening and SN-related cell therapy.

## Methods

hPSC maintenance and serotonin neuron differentiation were performed as we previously described [5, 6]. Detailed information for cell culture reagents were listed in Additional file 1: Table S1.

### Knock-in of EGFP-reporter cassette in *FEV* locus

The sgRNA was designed using the Benchling CRISPR Design Tool (https://www.benchling.com/crispr). Briefly, the 200-bp regions flanking the stop codon of the human FEV gene were analyzed to identify appropriate targeting sites, and one pair of sgRNA were selected. Then the synthesized sgRNA oligo (Genewiz, Suzhou, China) was annealed and ligated into plasmid PX330 (Addgene #42230) to form the targeting vector. As for the donor vector, the HAL (~ 1.7 kb upstream of the *FEV* stop-codon) and the HAR (~ 1.5 kb downstream from *FEV* stop-codon) were amplified from human genomic DNA (gDNA). The construction of pUC19-T2A-EGFP-CMV-PuroR reporter plasmid was constructed as we previously described. Subsequently, the HAL and the HAR were assembled into the reporter plasmid by seamless cloning (NovoRec® plus One step PCR Cloning Kit, NovoProtein, Shanghai, China). Shanghai, China). Sanger sequencing was performed to verify in frame positioning of the insertion cassettes. The sequence of sgRNA oligo was listed in Additional file 1: Table S2.

SgRNA-Cas9 plasmid (5 μg) and FEV-T2A-EGFP plasmid (10 μg) were electroporated to 0.5 million hPSC cells (Gene Pulser Xcell, Bio-Rad, 250 V, 250 μF, ∞ Ώ). Subsequently, the cells were transferred onto the Ƴ-ray-treated mouse embryonic fibroblasts (MEF)-coated 6-well plates containing human embryonic stem cell medium (DMEM/F12, 20% Knockout™ SR, 1 × 10^–4^ M 2-mercaptoethanol, 1 × nonessential amino acids, 1 × GlutaMAX) supplemented with 20 ng/ml bFGF and 10 μM Y27632. After 24 h of recovery, the medium was changed every two days with MEF-conditioned medium supplemented with 20 ng/ml bFGF and 0.4 μg/ml puromycin for 5 days. The puromycin-resistant clones were selected and expanded on Matrigel-coated 48-well plate with mTeSR1™ medium.

### PCR screening

Upon attaining approximately 80% confluence, the selected clones were subsequently expanded into 48-well plates. The gDNA of these clones was extracted using the Kapa gDNA extraction kit. To conduct PCR screening, three pairs of primers (5FR, 3FR and FR) primers were designed. The resulting PCR products were validated by gel electrophoresis and sequencing. Detailed sequences for the 5FR, 3FR, and FR primers can be found in Additional file 1: Table S2 for reference.

### Investigation of off-target sites

The top 10 potential off-target sites were analyzed by Cas-OFFinder online tool (www.rgenome.net/cas-offinder). Fragments with the length of 500 ~ 1000 bp surrounding these sites were amplified from the gDNA of the FEV-reporter cell line and then sequenced. The information of the top 10 potential off-target sites was listed in Additional file 1: Table S3.

### Immunocytochemistry

Immunocytochemistry was performed as we previously described [5]. Briefly, cells were fixed with 4% paraformaldehyde (PFA) for 30 min, followed by 3 consecutive washes with DPBS. Then the cells were blocked with 10% donkey serum and permeabilized with 0.2% Triton X-100 in DPBS for 30 min. Primary antibodies were applied to the cells and incubated overnight at 4 °C. After washing with DPBS for 3 times, the cells were incubated with the secondary antibodies and DAPI for 45 min at room temperature. Finally, the coverslips were rinsed and mounted onto microscope slides using anti-fade fluorescence mounting medium (Southern Biotech, 0100-01, Birmingham, USA). Imaging was carried out using confocal microscopes (Leica SP8 and Olympus FV3000).

### Teratoma formation assay

Approximately 5–6 × 10^7^ FEV-reporter hPSCs (H9-Clone#7) were dissociated using ReLeSR and resuspended in 120 μL Matrigel. The cells were then subcutaneously injected into the severe combined immunodeficient (SCID) mice. After an engraftment period of 8 weeks, the teratomas were harvested from the mice and fixed in 4% PFA overnight. Subsequently, the teratomas were immersed in a 30% sucrose solution to facilitate complete dehydration. Finally, the teratomas were embedded in OCT for cryosectioning and processed for hematoxylin and eosin (H&E) staining following the manufacturer's instructions.

### Fluorescence-activated cell sorting

The cells were dissociated using accutase and subsequently passed through a 40 µm cell strainer to obtain single cells. The filtered cells were then subjected to centrifugation (1200 rpm, 3 min). The collected cells were resuspended in NDM, supplemented with 10 μM Y27632. FACS was performed under optimized conditions for the sorting of neuronal cells (75 μm nozzle; a sheath fluid pressure of 20–25 pounds/square inch; acquisition rate of 1000–3000 events/second). The EGFP + cells were collected and subsequently reseeded on coverslips or 96-well plates coated with PO/Matrigel/laminin in NDM supplemented with 10 μM Y27632.

### Electrophysiology

Two weeks after sorting and reseeding, the EGFP + cells that had been purified were subjected to perfusion with a balanced salt solution (127 mM NaCl, 1.9 mM KCl, 2.2 mM CaCl_2_, 1.2 mM KH_2_PO_4_, 26 mM NaHCO_3_, 1.4 mM MgSO_4_, and 10 mM glucose, pH 7.3). The recording electrodes, fashioned from glass micro-capillaries, were filled with an internal fluid composed of 20 mM KCl, 10 mM Na^+^-HEPES, 121 mM K^+^-gluconate, 10 mM BAPTA, 4 mM Mg^2+^-ATP, and maintained at a pH of 7.2. These electrodes exhibited an electrical resistance within the range of 3 to 5 MΩ when immersed in the internal fluid bath. Voltage was stepped from -40 mV to + 30 mV in 5 mV increments to trigger Na + /K + currents. The data were recorded using the DigiData-1440A converter and processed with Clampex 10.2 software (Molecular devices), with subsequent analysis conducted using Clampfit 10.2 software.

### Detection of extracellular 5-HT

Three weeks following the sorting and reseeding procedure, the EGFP + cells that had been purified were cultured in 65 μL of NDM within 96-well plates, with a density of 1 × 10^4^ cells/well. Subsequently, Escitalopram oxalate (EO) (10 μM), a selective serotonin reuptake inhibitor, was added to the respective wells, and the cells were cultured for 24 h. Following this incubation period, the supernatants were collected. To measure the concentration of serotonin, enzyme-linked immunosorbent assay (ELISA) was conducted in accordance with the manufacturer's provided instructions.

### Smart RNA-sequencing

Sequencing libraries were generated from FEV reporter cell line (H9-Clone#7) (n = 3) by using the NEB Next Single Cell/Low Input RNA Library Prep Kit for Illumina. The library preparations were sequenced on Illumina NovaseqTM 6000 sequence platform and a total of 50 million 2 × 150 bp paired-end reads were generated. All reads were aligned to the human genome by HISAT2. Data were analyzed by online OmicStudio tools (https://www.omicstudio.cn) and Hiplot Pro (https://hiplot.com.cn). GO annotation of the DEGs was carried out using the Metascape database. DEGs were mapped to the search tool (https://string-db.org) for retrieval of interacting genes to acquire protein–protein interaction (PPI) networks. Visualization of the PPI network was performed by Cytoscape 3.9.1 software.

### mRNA extraction and qPCR

Total RNA was extracted using SteadyPure quick RNA extraction kit (Accurate Biology, China) and diluted in RNAase free water. One μg of RNA was reverse transcribed to cDNA using HiFiScript gDNA removal RT MasterMix (CW BIO, China). qPCR was performed using the SYBR Green Mix (Bio-Rad, USA) in a BioRad CFX96 Thermal Cycler (Bio-Rad, USA). Housekeeping gene GAPDH was used to normalize mRNA levels between different samples. Primer sequences are listed in Additional file 1: Table S2.

### Statistical analysis

Statistical analysis was performed using GraphPad PRISM 8 (GraphPad Software, San Diego, CA, USA). Unpaired student’s *t*-test was used to compare the difference between two groups. All data were expressed as the mean ± the standard error of the mean (SEM). *P < 0.05, **P < 0.01 and ***P < 0.001 were considered to be significant.

## Results

### Generation of FEV reporter hPSC

**Fig. 1:**
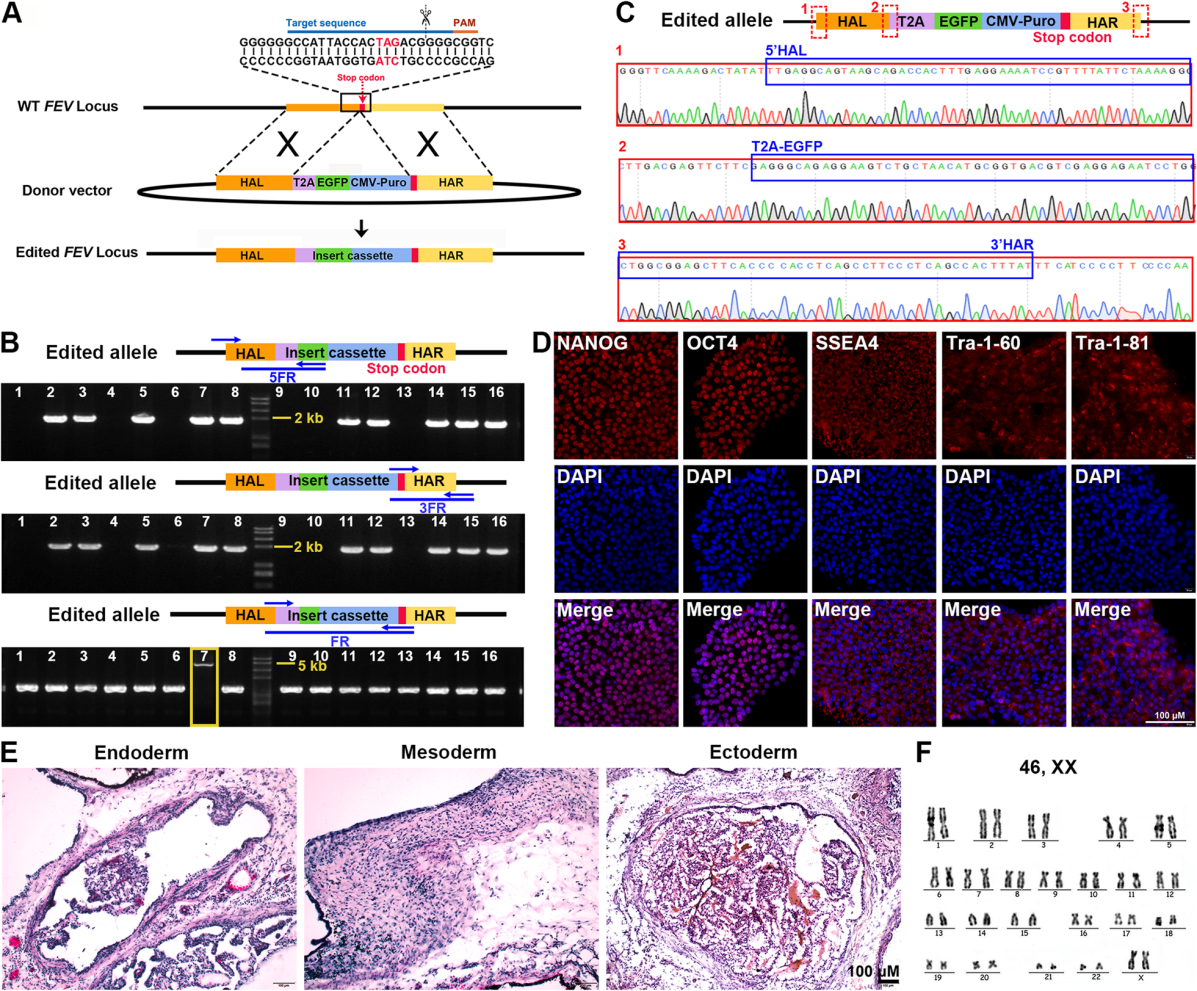
Generation of FEV reporter hPSC. **A** Schematic diagram for strategy of CRISPR-Cas9-mediated knockin of reporter cassette into human *FEV* locus. **B** Genotyping of the edited hPSC (H9) clones with the primer sets (5FR, 3FR, FR) to identify the edited homozygous clone. (The 5FR and 3FR primer sets spanned the nucleotides outside the homologous arm to the internal reporter/selection cassette, aiming to verify the integration of the insertion cassette into the *FEV* locus; the FR primer set, flanking approximately 1500 bp of the stop codon of WT *FEV* gene, was designed to distinguish between homozygous and heterozygous clones) **C** Representative Sanger sequencing chromatograms for the homozygous Clone#7. **D** Immunofluorescence staining of the pluripotency markers (OCT4, NANOG, SSEA4, Tra-1-60, Tra-1-81) for the FEV-EGFP reporter cell line (H9-Clone#7). **E** H&E staining for the FEV-EGFP reporter cell line (H9-Clone#7)-derived teratoma. **F** Karyotyping analysis for the FEV-EGFP reporter cell line (H9-Clone#7). Scale bar for **D**, **E** 100 μm

To identify premature human SN, an FEV-EGFP reporter cassette was introduced to the coding region immediately upstream of the stop codon of the endogenous *FEV* gene by CRISPR/Cas9-mediated homologous recombination (Fig. 1A). Edited hPSC clones were screened by PCR assay. As shown in Fig. 1B, clones (2, 3, 5, 7, 8, 11, 12, 14, 15, 16) exhibited two 2000 bp-band (5FR and 3FR), indicating the in-frame integration of the reporter cassette into the *FEV* locus. Clone 7 showed a 5000 bp-band (FR), indicating homozygous integration of the FEV reporter cassette. The top 10 potential off-target sites (Additional file 1: Table S3) were evaluated in the homozygous Clone 7 by Sanger sequencing, and no insertion/deletion (indel) mutation was detected in these sites, suggesting the high specificity of this gene editing system (Additional file 1: Figure S1). The homozygote reporter cell line (H9-Clone#7) was then selected for the entire study unless specially indicated. The Sanger sequencing showed the sequences of 5’-end junction, the T2A-EGFP junction and the 3’-end junction between the original *FEV* DNA sequence and the inserted sequence (Fig. 1C), indicating the in-frame integration of the reporter cassette. The FEV-EGFP reporter hPSC expressed pluripotent markers (Fig. 1D) and was able to form teratoma (Fig. 1E), indicating its pluripotency properties. Additionally, the engineered reporter hPSC showed normal karyotype (Fig. 1F). These data suggested that T2A-EGFP-CMV-puro cassette was correctly inserted into the endogenous *FEV* gene of the hPSC with high efficiency and specificity, and the FEV-EGFP reporter hPSC maintained pluripotency with the normal karyotype.

### Monitoring FEV expression during human SN differentiation via FEV reporter system

**Fig. 2:**
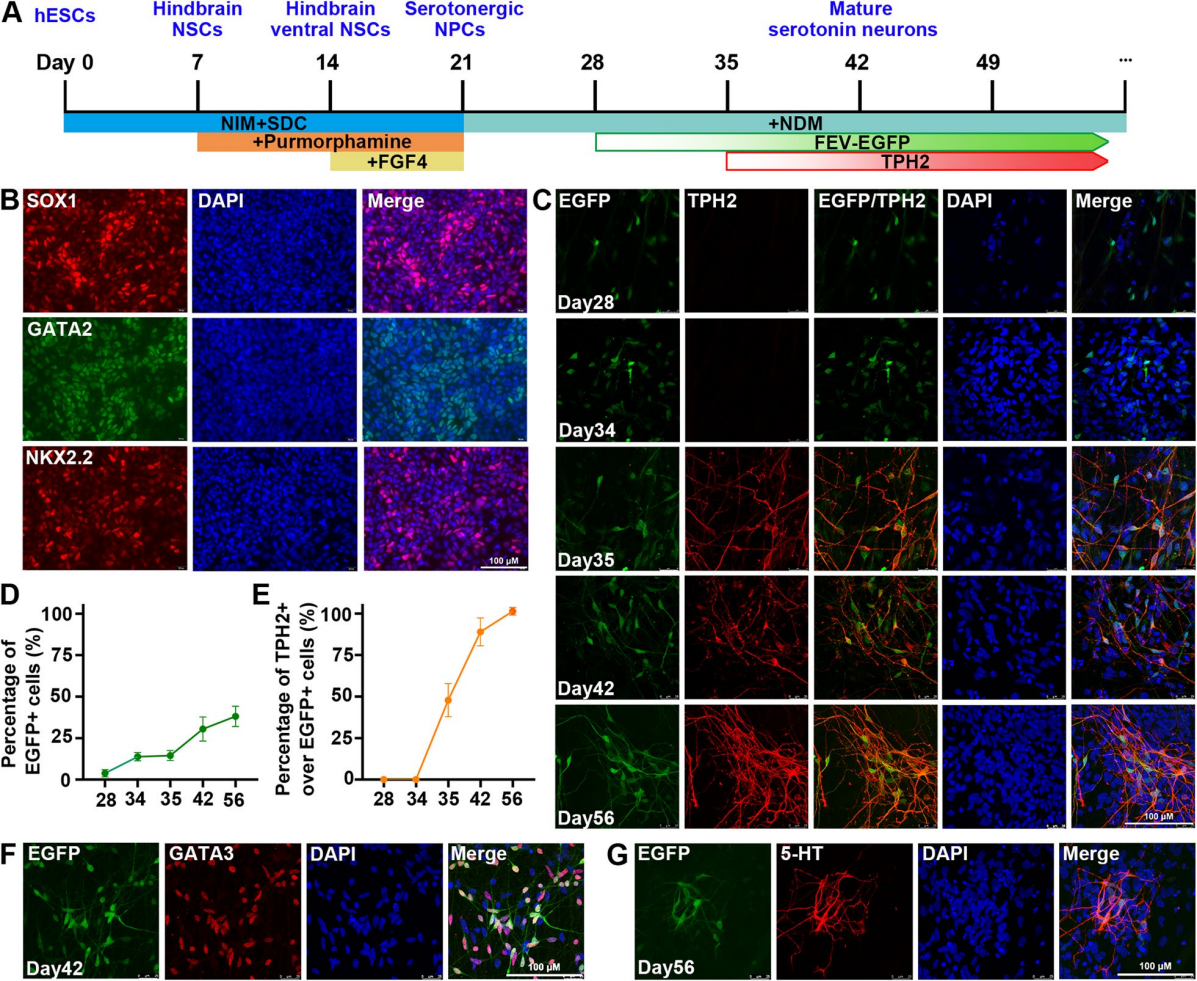
Investigation of the expression timing of FEV during SN differentiation. **A** Schematic diagram of the strategy to derive SN from the FEV reporter hPSC**. B** Immunofluorescence staining of SOX1, GATA2 and NKX2.2 to indicate reporter cell line (H9-Clone#7)-derived SNP at day 21 of differentiation. **C.** Immunofluorescence staining of EGFP and TPH2 to validate the expression time of FEV and TPH2. **D, E** Time course of FEV **(D)** and TPH2 **(E)** expression during SN differentiation process. **F** Immunofluorescence staining of GATA3 at day 42 of differentiation. **G** Immunofluorescence staining of EGFP and 5-HT at day 56 of differentiation. Scale bar for **B, C, F, G** 100 μm. n = 3 independent experiments. All data are presented as means ± SEM

In order to determine the timing of FEV expression, the FEV-EGFP reporter hPSC were differentiated into SN according to our established protocol [5] (Fig. 2A). Co-activation of WNT and SHH signaling pathways induced the reporter hPSC to differentiate into serotonergic neural progenitors (SNP) expressing SOX1, GATA2 and NKX2.2 (Fig. 2B). Subsequently, SNP continued to differentiate into SN. As shown in Fig. 2C–E, FEV expression initiated at day 28 of differentiation towards SN, preceding the emergence of TPH2 (a specific marker for mature SN) by about one week. Hence, we defined the FEV + /TPH2- cells as immature SN. The proportion of EGFP + cells was positively corelated to the duration of differentiation (Fig. 2C, D), suggesting a progressive upregulation of FEV expression accompanying the maturation of SN over time. At day 56, all of the EGFP + cells were co-labeled with TPH2 (Fig. 2C, E), indicating that FEV-EGFP specifically labeled the early-stage SN. Meanwhile, the EGFP + cells were also co-labeled with other typical serotonergic markers, such as GATA3 (Fig. 2F) and 5-HT (Fig. 2G), validating the serotonergic identity of FEV + cells. Additionally, the engineered reporter hPSC showed similar differentiation potential with the parental hPSC (H9 cell line, Additional file 1: Figure S2), indicating that the differentiation potential of the reporter hPSC was not compromised by the gene-editing process. Therefore, these data demonstrated that the onset of FEV expression occurred 7 days prior to the expression of TPH2, and FEV expression gradually increased with the maturation process of SN over time.

### Validation of the role of FEV as the specific marker for immature SN

**Fig. 3:**
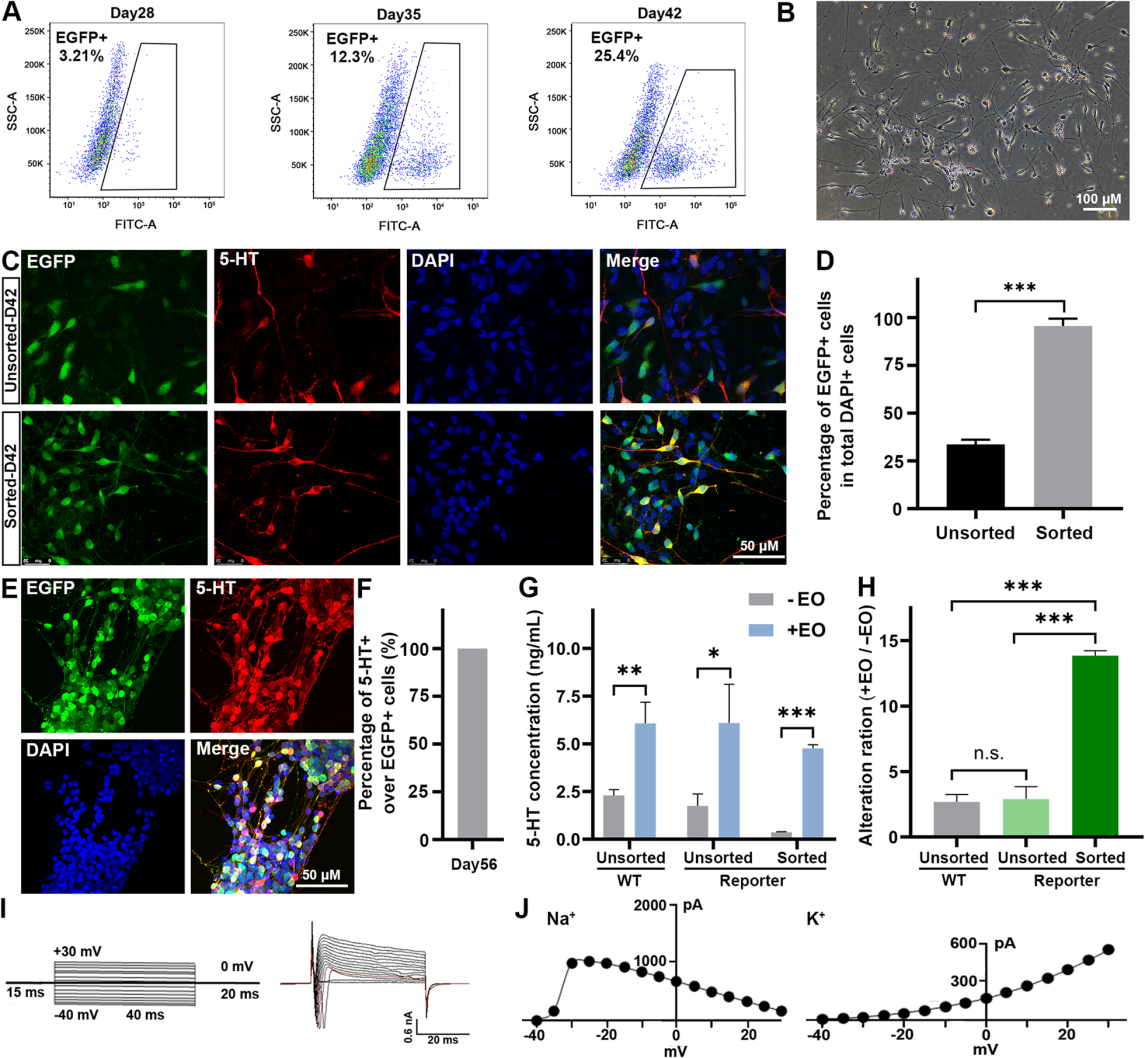
Purification and validation of FEV-EGFP reporter system-derived SN. **A** FACS profiles of FEV-EGFP reporter cell (H9-Clone#7)-derived cells during differentiation from day 28 to day 42. **B** Representative brightfield image of the purified EGFP + cells 16 h after FACS-based purification and reseeding. **C, D** Immunofluorescence staining **(C)** and quantification **(D)** of EGFP and 5-HT for unsorted and sorted SN at day 42 of differentiation. **E, F** Immunofluorescence staining **(E)** and quantification **(F)** of EGFP and 5-HT for sorted SN at day 56 of differentiation. **G** Extracellular 5-HT released by unsorted or sorted cells before and after treatment with EO. **H** Evaluation of the sensitivity of the pharmacological responses to EO by unsorted or sorted SN. **I** Input voltage program (protocol) and current traces evoked by 40-ms depolarizing voltage stepped from − 40 to + 30 mV in 5-mV increments. **J** Current–voltage curves for voltage-gated sodium and potassium currents (n = 6). Scale bar for **B**: 100 μm; scale bar for **C**,** E**: 50 μm. n = 3 independent experiments. All data are presented as means ± SEM. Statistical comparisons were performed by Student’s t test. *P < 0.05; **P < 0.01; ***P < 0.001, n.s.: no significance

Since the FEV-EGFP reporter system could faithfully indicate FEV expression in early-stage SN (Fig. 2), we next analyzed the FACS-purified EGFP + cells. With the increasing duration of differentiation, an increasing proportion of FEV-EGFP-positive cells could be obtained through FACS (Fig. 3A). The purified cells at day 35 of differentiation were reseeded onto poly-L-ornithine (PO)/laminin/Matrigel-coated coverslips, and exhibited rapid attainment of a healthy state and the extension of nerve fibers within 16 h following reseeding (Fig. 3B). Moreover, the percentage of EGFP + cells (95.63% ± 1.252%, n = 10) in the purified population was significantly higher than that (33.64% ± 0.796%, n = 10) in the mixed cell population (Fig. 3C, D), indicating that the FEV-EGFP reporter enabled us to purify early-stages SN from heterogeneous differentiation cultures in vitro. 3 weeks after reseeding (day 56), all of the EGFP + neurons were co-stained with 5-HT (Fig. 3E, F), indicating the role of FEV as a terminal selector for SN. Subsequent functional analysis was conducted on the purified FEV-EGFP-labeled mature SN (at day 56). These cells released 5-HT in response to escitalopram oxalate (EO, a selective serotonin reuptake inhibitor) (Fig. 3G, H). Additionally, when subjected to a 40-ms voltage step ranging from − 40 mV to + 30 mV, the FEV-EGFP-labeled SN (at day 56) exhibited characteristic inward Na + and outward K + currents (Fig 3I, J), indicating their functional properties as mature SN. Therefore, the FEV-EGFP reporter enabled us to obtain highly purified immature SN, which underwent maturation within 3 weeks and were capable of showing typical features of mature SN.

### Alterations of transcriptomic profiles during human SN differentiation process

**Fig. 4:**
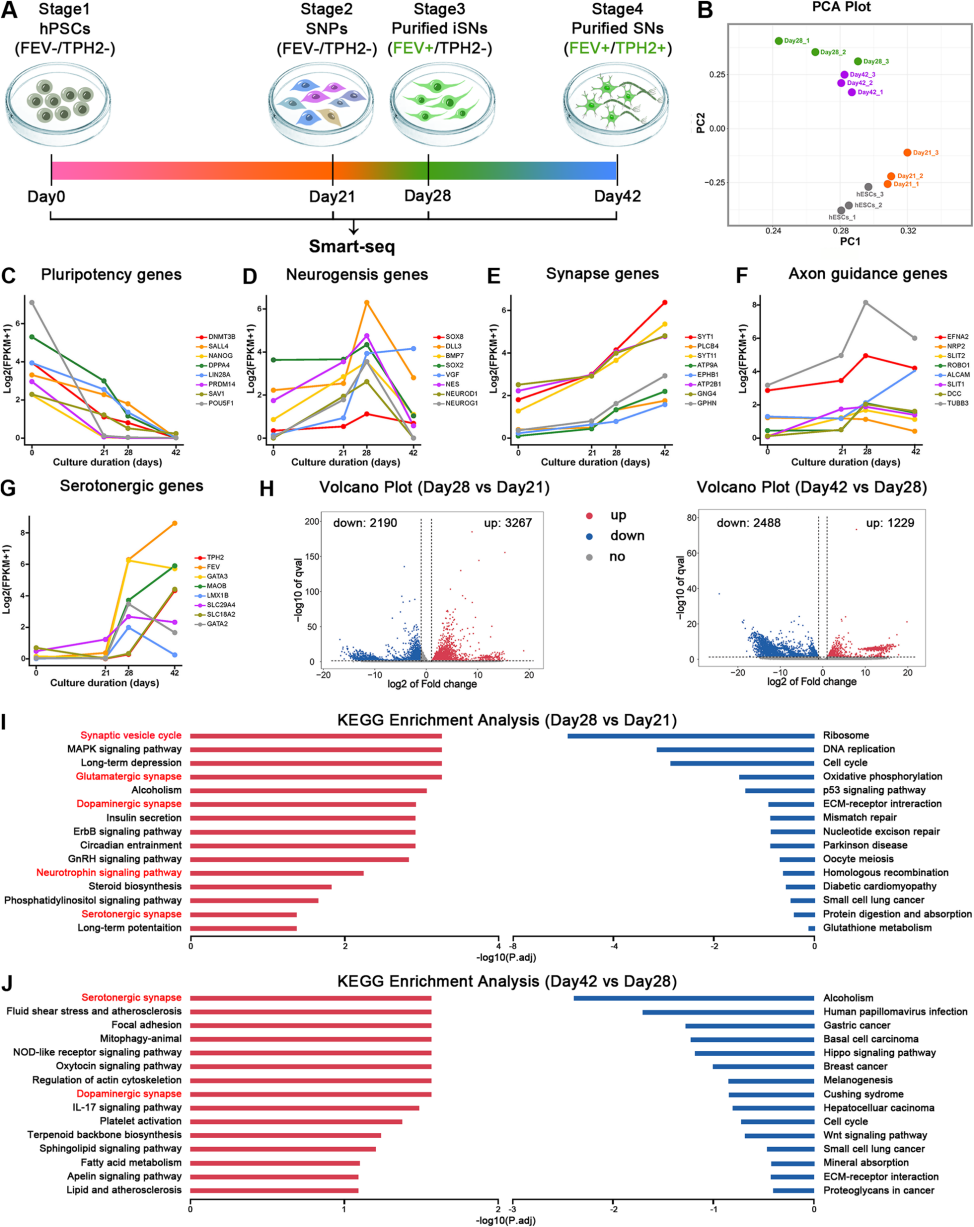
Analysis of transcriptomic profiles during human SN differentiation process. **A** The schematic of sample collection from four stages during SN differentiation for smart RNA-seq (Day0: hPSC stage; Day21: SNP stage; Day28: immature SN stage; Day42: mature SN stage). **B** Principal component analysis (PCA) score plot for the samples. **C–H** Gene expressions of pluripotency **(C)**, neurogenesis **(D)**, synapse **(E)**, axon guidance **(F)** and serotonergic genes **(G)** at 4 key stages during SN differentiation. **H.** Volcano plots of the DEGs. The up-regulated, down-regulated and unchanged metabolites are presented by red, blue, and gray dots, respectively. **I, J** The functional classification of the DEGs (**I**: day 28 vs. day 21; **J** day 42 vs. day 28) based on Kyoto Encyclopedia of Genes and Genomes (KEGG) pathway analysis

To comprehensively reveal the dynamic changes of transcriptomic profiles during human SN differentiation, we investigated 4 key stages of the differentiation process by RNA-seq analysis (Fig. 4A). Cells at Stage 3 and 4 were purified by FACS via FEV-EGFP reporter and TPH2-EGFP reporter respectively. Principal-component analysis (PCA) remarkably separated the samples into 4 clusters (Fig. 4B). The expression of the key differentiation stage genes, including "pluripotency genes", "neurogenesis genes", "synapse genes", “axon guidance genes” and "serotonergic genes", were indicative of their expression patterns during specific developmental stages (Fig. 4C–G). Volcano plots showed thousands of differentially expressed genes (DEGs) expressed in the individual key stage during differentiation (Fig. 4H). Kyoto Encyclopedia of Genes and Genomes pathway (KEGG) enrichment analysis was performed to identify functional categories of the DEGs in the individual key stage of differentiation. The upregulated DEGs at day 28 and day 42 were enriched in synapse- and serotonergic-associated pathways, indicating the determination of serotonergic fate with FEV expression at day 28 and progressive neuronal maturation at day 42 (Fig. 4I, J). These data revealed dynamic changes of transcriptomic profiles during human SN differentiation.

### Identification of the potential key molecules crucial for SN fate specification

**Fig. 5:**
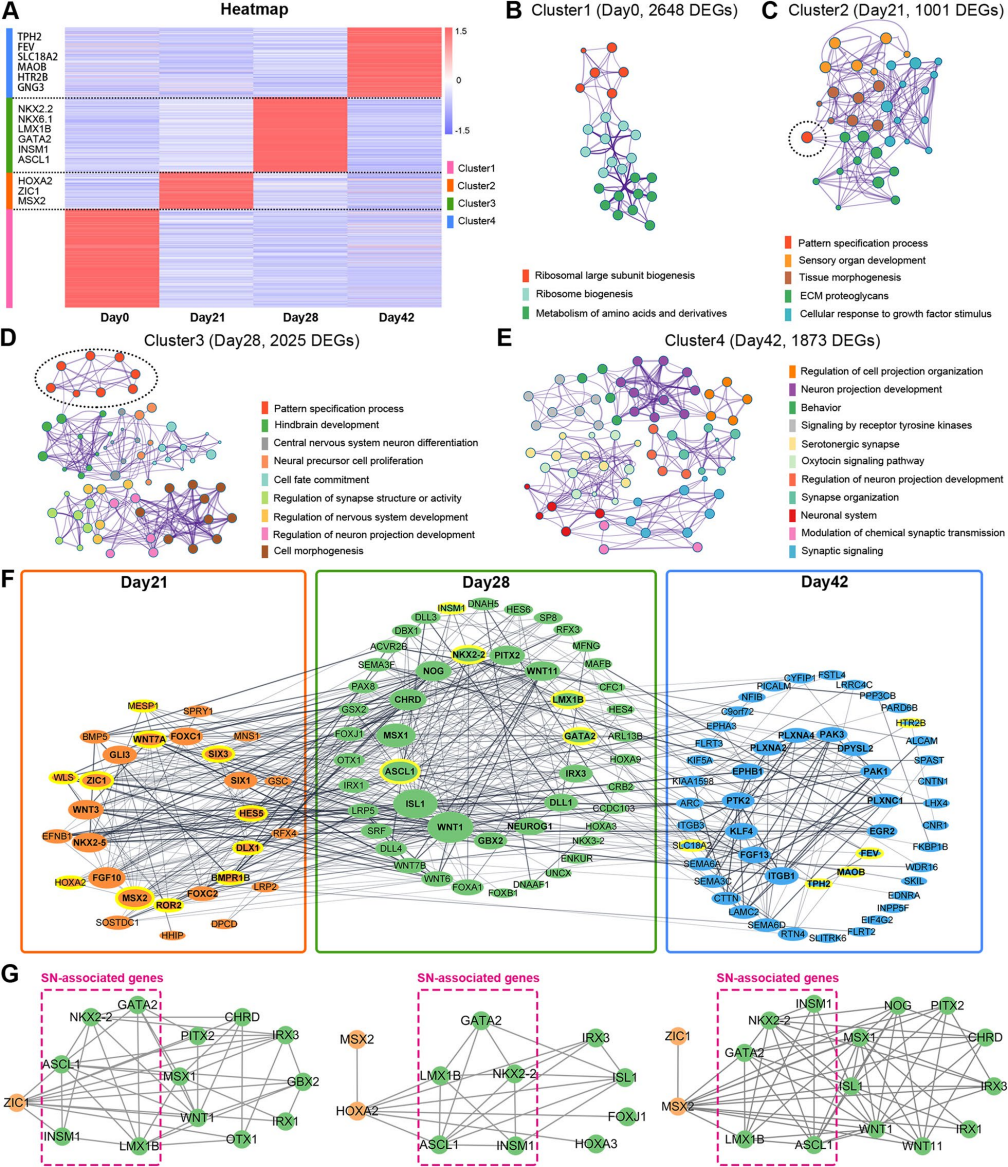
Identification of the potential molecules responsible for serotonergic fate determination. **A** Heatmap of the DEGs exclusively expressed at each key differentiation stage. **B–E** Gene ontology (GO)-based functional analysis of the marker genes exclusively expressed by **(B)** cluster1, **(C)** cluster2, **(D)** cluster3 and **(E)** cluster4.** F** Protein-to-protein interaction (PPI) networks of the DEGs enriched in GO terms shown in **B–E**. **G** PPI networks involving the DEGs (day 21) having interactions with SN-associated genes expressed at day 28 of differentiation

While we demonstrated that the cells committed to a serotonergic fate as early as day 28 of the differentiation process (Figs. 3, 4), the mechanisms responsible for initiating serotonergic fate determination before this time point remain elusive. Therefore, the K-means clustering algorithm was employed to analyze the DEGs expressed at each key differentiation stage (Fig. 5A): we identified that immature SN (at day 28) expressed several key transcription factors, including *NKX2.2*,* NKX6.1*, *LMX1B*, *GATA2*, *INSM1* and *ASCL1*; in addition to the typical mature serotonergic markers, *SLC18A2*, *MAOB* and *HTR2B*, mature SN (at day 42) expressed high level of transcription factor *FEV*, indicating a potential role for FEV in facilitating and sustaining the maturation of human SN. GO enrichment analysis was performed for functional analysis of the marker genes shown in Fig. 5A. DEGs of cluster1 (day 0) exhibited significant enrichment in “ribosome biogenesis” pathway, indicating an increased demand for protein synthesis essential for cellular growth and differentiation (Fig. 5B). Compared to cluster2 (day 21), cluster3 (day 28) showed an augmented enrichment in the “pattern specification process” term. Moreover, this stage exhibited enrichment in terms such as neural precursor cell proliferation, cell fate commitment, regulation of synapse structure or activity, and regulation of neuron projection development (Fig. 5C, D). These findings suggested that the initiation of serotonergic fate specification might occur as early as day 21. Finally, the DEGs of cluster4 were highly enriched in “neuron/cell projection” and “synapse”-associated pathways (Fig. 5E), suggesting an active process of establishing synaptic connections and facilitating communication within the neighboring nervous system, which is a characteristic hallmark of neuronal maturation. To elucidate the crucial molecular events occurring during SN differentiation, we constructed interaction networks for DEGs enriched in the critical GO terms as presented in Fig. 5C–E, utilizing a protein–protein interactions (PPIs) degree analysis approach. As shown in Fig. 5F, the top 15 hub genes, ranked based on degree (the number of connections or a node possesses), were listed in the inner circle; and the genes of interests or those previously identified as critical for SN development were highlighted by yellow circle. This network emphasized the importance of transcription factors, including* ASCL1*, *NKX2.2*, *LMX1B*, *GATA2* and *INSM1*, for the maturation of SN; furthermore,* ZIC1*, *HOXA2* and *MSX2* exhibited remarkable interactions with the SN-associated genes, indicating their pivotal roles in the differentiation process spanning from day21 to day28 (Fig. 5F, G). Then qPCR was performed to validate the expression levels of the hub genes and SN-associated genes: The qPCR data (Additional file 1: Figure S3) generally aligned with the RNA-seq data (Fig. 5A, F), although there were some slight discrepancies.

### Identification of the crucial genes and signaling pathways based on weighted gene co-expression network analysis (WGCNA)

**Fig. 6:**
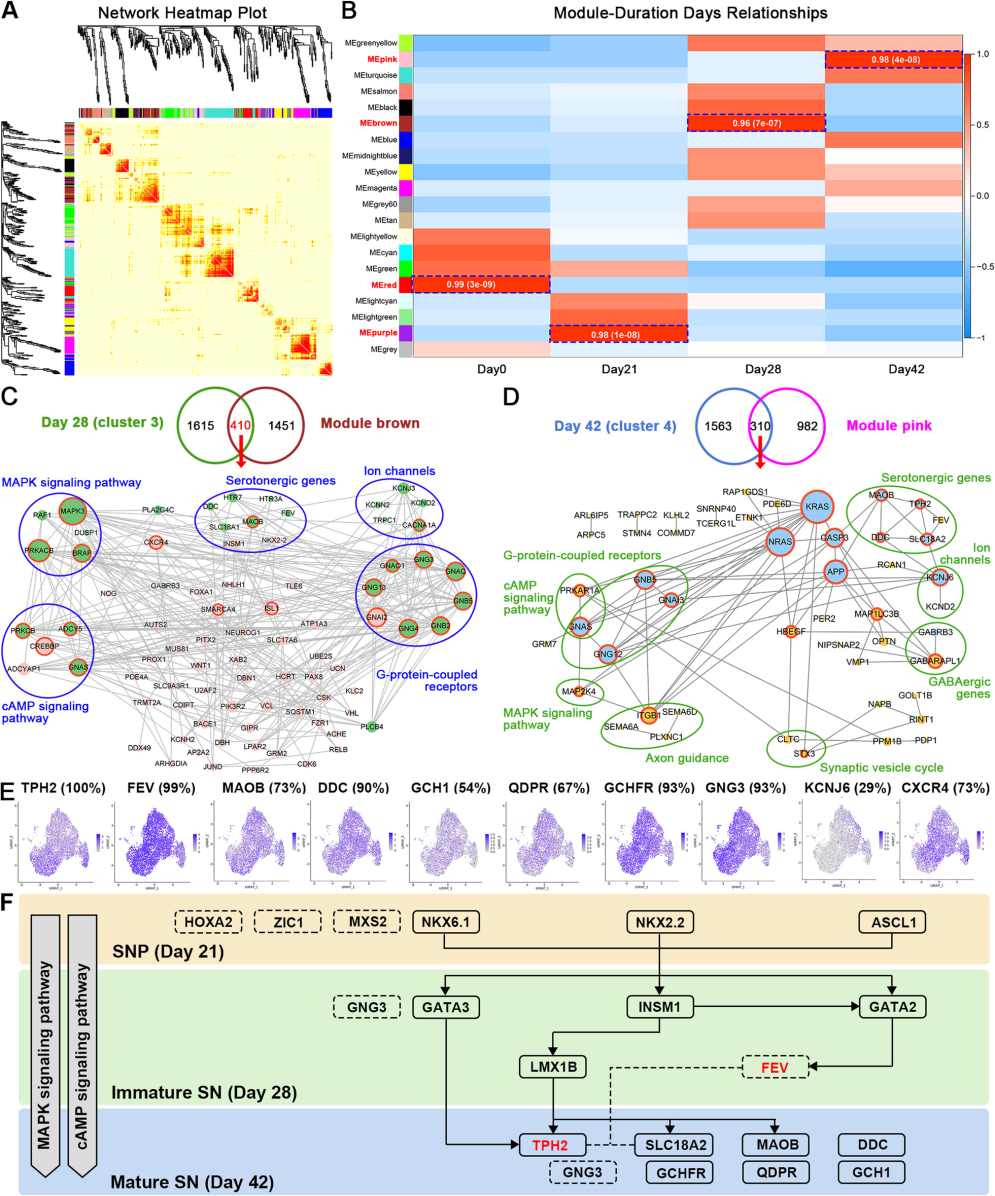
Exploration of the key signaling pathways during SN differentiation. **A** Heatmap plot of topological overlap matrix (TOM) in the gene network. Rows and columns correspond to single genes, light color represents low overlap and progressively darker red color represents higher overlap. Blocks of darker colors along the diagonal are the modules.** B** The correlation of DEGs in modules across key stages of SN differentiation. **C** PPI analysis of the overlapping DEGs exclusively expressed on day 28 and in module brown.** D** PPI analysis of the overlapping DEGs exclusively expressed on day 42 and in module pink. **E** Expression patterns of the serotonergic-associated genes across individual serotonin neurons presented as UMAP plots. **F** Schematic of the signaling pathways and molecules that paly essential roles during SN differentiation

Instead of a narrow focus on DEGs, WGCNA efficiently clusters the highly interconnected genes into diverse modules, enabling a comprehensive exploration of correlations between the modules and traits of interest. WGCNA was employed to construct the co-expression network among DEGs, adhering to the scale-free topology criteria: the soft-thresholding power β was set at 17 to reach the criteria (scale-independence > 0.85, average connectivity < 100) for the establishment of a scale-fee co-expression network (Additional file 1: Figure S4A, B). 20 modules were identified (Additional file 1: Figure S4B) and the heatmap plot of topological overlap matrix (TOM) of all genes was shown in Fig. 6A. Modules colored in red, purple, brown and pink showed the strongest correlations with the 4 key differentiation stages, respectively (Fig. 6B). To validate the accurate identification of modules, these modules were investigated again by calculating the mean absolute gene significance (GS) value for genes within each module. Notably, the GS-MM scatterplot for the brown and pink modules showed that the GS value was remarkably associated with the MM value (brown module: cor = 0.92, *p* < 1e−200; pink module: cor = 0.95, *p* < 1e−200) (Additional file 1: Figure S5), indicating the strong correlation of the two modules with the pivotal differentiation stages of SN. The gene set exclusively expressed on either day 28 or day 42 was intersected with the gene set of the brown or pink module respectively; then PPI analysis was conducted on the resulting two intersected gene sets. The hub genes of the two differentiation stages (day 28 and day 42) were identified to be associated with the cAMP and MAPK signaling pathways (Fig. 6C, D), underscoring the pivotal roles of these signaling pathways during the later stage of SN differentiation. To delineate the key molecules and signaling pathway networks involved in the differentiation of SN, an analysis was conducted using our previously published human SN single-cell RNA sequencing (scRNA) dataset [6] (GEO dataset: accession number GSE232698). Except for the well-known genes associated with serotonin synthesis and metabolism, including TPH2, FEV, MAOB, DDC, GCH1, QDPR and GCHFR, it was identified that 93% of mature human SN expressed GNG3 (Fig. 6E), which was also highly expressed in immature SN at day 28 of differentiation (Fig. 6C). Based on the above findings, the regulatory molecular network governing SN differentiation was exposed for the first time (Fig. 6F).

## Discussion

In the present study, we successfully established an FEV-EGFP reporter system with insertion of EGFP into the endogenous human *FEV* locus following the coding sequence through CRISPR/Cas9-mediated technology (Fig. 1, Additional file 1: Figs. S1 and S2). Consistent with the animal study which reported that mouse *Pet1* (orthologous to human *FEV*) exhibited the onset of expression preceding the emergence of 5-HT by a half day [10], the FEV-EGFP expression occurred one week before the initiation of TPH2 expression and persisted without diminishment in human SN (Fig. 2C-G). Some other studies also established protocols to generate human SN in vitro: (1) Human primary fibroblasts were directly converted to induced SN by overexpression of several SN-associated genes [14, 15]. In these two studies, the expression timing of FEV during SN reprogramming was unclear since FEV was overexpressed to induce the conversion toward SN; (2) Lu et al. established the protocol to differentiate human pluripotent stem cells towards rostral SN [4], and Valiulahi et al. [16] generated human caudal SN by adding retinoic acid (RA) based on the differentiation protocol established by Lu et al. However, they only reported FEV expression as a serotonergic marker during SN differentiation process without investigating the initial expression timing of FEV. Hence, by leveraging the advantages of the FEV reporter system, we can observe live cells expressing FEV via EGFP during the whole development process, enabling us to precisely monitor the onset of FEV expression during human SN development. Compared to the TPH2 reporter system [5], the FEV reporter system showed advantage of detection of both postmitotic FEV + /TPH2- immature SN and FEV + /TPH2 + mature SN (Fig. 2C), which helped to connect the SNP stage with the mature SN stage via highly-purified immature SN for subsequent transcriptomic analysis.

Previously, we investigated the transcriptomic profiles of cells at 3 differentiation time points (0, 21, 42 days) during human SN differentiation [5]. However, it is worth noting that a substantial 21-day interval existed between day 21 and day 42, which could potentially result in the oversight of important genes crucial for serotonergic fate determination. Here we employed the FEV-EGFP reporter system to derive highly-purified immature SN at day 28 of differentiation, and all of the FEV-positive cells were matured into TPH2-postive SN at day 56 of differentiation, validating its role as a terminal selector for SN. However, the presence of other molecules that predate FEV expression and function as serotonergic fate determinant remains uncertain. Subsequently, we conducted a comprehensive comparative analysis of the transcriptomic profiles of cells at four key stages of differentiation (PSC, SNP, postmitotic immature SN and mature SN) (Fig. 4A), holding potential to reveal the essential transcriptional signaling pathways and molecules responsible for serotonergic fate specification. Indeed, KEGG analysis exposed that the DEGs of purified FEV-positive cells (at day 28) were highly enriched in serotonergic synapse-associated pathways, indicating that the cells had adopted serotonergic fate as early as day 28 of differentiation. Notably, our analysis revealed the identification of approximately 2025 genes distinguished by their exclusive high expression levels on day 28 (Fig. 5A). Therefore, conducting a comparative analysis of DEGs at day 21 and 28 could provide insights into the earlier and essential molecular events that govern serotonergic fate specification.

The unbiased hierarchical clustering analysis exposed that the immature human SN (day 28) exhibited expression of several pivotal transcription factors, including *NKX2.2*, *NKX6.1*, *LMX1B*, *GATA2*, *INSM1*, and *ASCL1* (Fig. 5A, F). This observation aligned with the findings in mouse studies [17], underscoring the essential role of these transcription factors in SN development. Moreover, through the KEGG analysis of the marker genes of each key stage, cluster3 showed an augmented enrichment in the “pattern specification process” term in comparison to cluster2 (Fig. 5C, D), indicating the initiation of serotonergic fate specification may occur as early as day 21. We observed significant interaction between the hub genes shown in day 21 and day 28 of differentiation. A set of hub genes shown at day 21 of differentiation, including* HOXA2*, *ZIC1*, and *MSX2*, showed intimate interactions with the SN-associated genes (*NKX2.2*, *GATA2*, *LMX1B*, *INSM1*, *ASCL1*) expressed at day 28 of differentiation (Fig. 5F).

As the major components of the raphe nuclei, SN are born in the hindbrain rhombomere 1–8 region [18]. The caudal *Hoxa2* gene plays a crucial role in anterior–posterior patterning [19]. Moreover, accumulating evidence identified high expression of HOXA2 by neural progenitors during human SN differentiation process [4, 6, 16], indicating the crucial role of HOXA2 during SN differentiation. *Msx1/Msx2* mutant mice showed impaired patterning of cranial neural crest and altered expression of hindbrain marker genes, indicating that MSX2 might be crucial for the establishment of rhombomere identity [20]. Previously, we proved that retinoid acid (RA) signaling was crucial for SN differentiation by promoting caudalization [6]. Interestingly, *Zic1* was identified to regulate production and transport of RA [21], implying its role in SN differentiation by regulating anterior–posterior patterning. Despite extensive research, there is currently no conclusive evidence demonstrating the involvement of these genes in SN differentiation, nor their potential to serve as markers for earlier-stage SNP. Consequently, genetic manipulation of these genes and the subsequent analysis of the differentiation trajectory within the SN lineage at the single-cell level offer a promising avenue for elucidating their functions and for evaluating their suitability as markers for earlier-stage SNP. It is noteworthy that these genes do not exhibit a specific expression pattern identical to that of *FEV*. Therefore, the detailed expression patterns of these candidate genes should be validated at single cell level. Subsequently, a precise targeting of SNP could be achieved though the coordinated expression of a panel of genes.

*KCNJ6* was identified to be exclusively expressed by mature SN when compared to other differentiation stages (Fig. 6D). According to scRNA-seq data, 29% of human SN were observed to express *KCNJ6* (Fig. 6E). *KCNJ6*, encoding a potassium channel subunit 2 (GIRK2), is implicated in the modulation of reward-related brain processes [22], cognitive function [23] and pain sensitivity [24]. Interestingly, SN in the central nervous system (CNS) has been recognized as the only resource for the neurotransmitter serotonin which functions to regulate rewards [25], pain sensation [26] and cognition [27]. The biological role of *KCNJ6*, which exhibited high expression in one cluster of human SN (Fig. 6E), remains unclear. Future investigations into cellular functional phenotypes via genetic manipulation may provide insights into its function within SN.

SN displayed elevated expression of *CXCR4* from day28 of differentiation (Fig. 6C), with approximately 73% of SN expressing *CXCR4* at the mature stage (day42 of differentiation) (Fig. 6E). This result is consistent with the previous study, which reported that over 70% of rat SN in the dorsal raphe nucleus co-localize with CXCR4 [28]. Moreover, CXCL12, the ligand of CXCR4, could modulate the electrophysiological properties of SN and 5-HT secretion [28]. Indeed, CXCL12/CXCR4 axis plays pivotal roles in CNS during the development process as well as in the later life. In addition to its classical roles in the recruitment of immune cells and the migration of neural precursor cells, recent studies suggested that CXCL12/CXCR4 signaling also modulated motor axon guidance [29], synaptic plasticity [30], neuronal survival [31] and neurotransmitter (glutamate [32], 5-HT [28]) release. Therefore, further exploration of the relationship between the CXCL12/CXCR4 axis and the function of human SN may offer insights to understand the role of immune system in the development and pathology of serotonergic system.

The elucidation of the pivotal genes and the transcriptional profiles facilitates a deeper understanding of the developmental mechanisms for human SN. This, in turn, aids in unraveling the challenges restricting the SN differentiation efficiency and the difficult tasks for SN tracing. On the other hand, understanding transcriptional profiles of human SN enabled us to explore the intricate correlations between the SN and a spectrum of neuropsychiatric disorders. For example, SN are well-documented to be involved in setting the seizure threshold [33], but the underlying mechanism is largely unknown. Schwindinger. et al. reported the mice with G Protein Subunit Gamma 3 (GNG3) deficiency showed increased susceptibility to seizures, ultimately resulting in a reduced lifespan [34, 35]. In the present study, *GNG3* was identified to be highly expressed by SN during the differentiation process from day 28 to day 42 (Fig. 6C, E). Therefore, genetic manipulation of *GNG3* in human SN might help to elucidate the underlying mechanism of SN-associated seizure development.

Limitation of the reporter system: recent animal studies reported that transplantation of SN-enriched rodent embryonic raphe nucleus could improve locomotor and cardiovascular function following spinal cord injury (SCI) [36, 37], indicating the therapeutic potential of SN-based cell therapy on SCI. However, transplantation of stem cell-derived fully matured neurons could be challenging due to their intricately branching dendrites and axons, as well as their limited capacity for survival and integration within the host tissues. Therefore, compared to fully differentiated mature neurons, neural progenitors are commonly used for transplantation due to their notable survival rates following transplantation. However, hPSC-derived neural progenitors usually contain a variety of cell types with multiple fate potentials, leading to that only a small proportion of cells with serotonergic fate can differentiate into mature SN with therapeutic effect following transplantation into the brain. Thus, the ideal cells for transplantation would be highly-purified neural progenitors with serotonergic fate. It is worth-mentioning that FEV-labeled cells (day 28) initially exhibited a neuron-like morphology characterized by a limited number of dendrites and axons (Fig. 2C), hampering its application for transplantation (data not shown). To address this issue, identification of a novel serotonergic marker which is expressed earlier than FEV and establishment of a reporter system based on this novel marker might be a promising solution. Fortunately, the present FEV-EGFP reporter system enabled us to derive highly-purified immature SN for investigating the transcriptional alteration of the cells during differentiation towards SN, with a particular focus on the identification of potential earlier markers indicative of SNP.

## Conclusions

In conclusion, this study successfully established a human FEV reporter system using CRISPR/Cas9 gene-editing technology. We investigated the precise timing of FEV expression in human SN. Furthermore, for the first time, we exposed the developmental transcriptomic profiles of human SN via FEV reporter system, which will further our understanding for the development process of human SN and help the SN-related research and application.

### Supplementary Information

**Additional file 1**: Supplementary Figures and Tables.

## Data Availability

RNA-seq data have been deposited in the Gene Expression Omnibus Database (GEO) under accession number GSE246547 (https://www.ncbi.nlm.nih.gov/geo/query/acc.cgi?acc=GSE246547).
