# Supplementary material for: Transcriptomic profiling across human serotonin neuron differentiation via the FEV reporter system
Source: Stem Cell Res Ther. 2024 Apr 19;15:107. doi: 10.1186/s13287-024-03728-x (PMC11027224; doi:10.1186/s13287-024-03728-x)
Supplement: Supplementary file 1 — Additional file 1: Supplementary Figures and Tables. [file 13287_2024_3728_MOESM1_ESM.pdf]

Supporting Information

Transcriptomic Profiling across Human Serotonin Neuron Differentiation via the FEV Reporter System

Yingqi Li, Jinjin Duan, You Li, Meihui Zhang, Jiaan Wu, Guanhao Wang, Shuanqing Li, Zhangsen Hu, Yi Qu, Yunhe Li, Xiran Hu, Fei Guo, Lining Cao*, Jianfeng Lu*

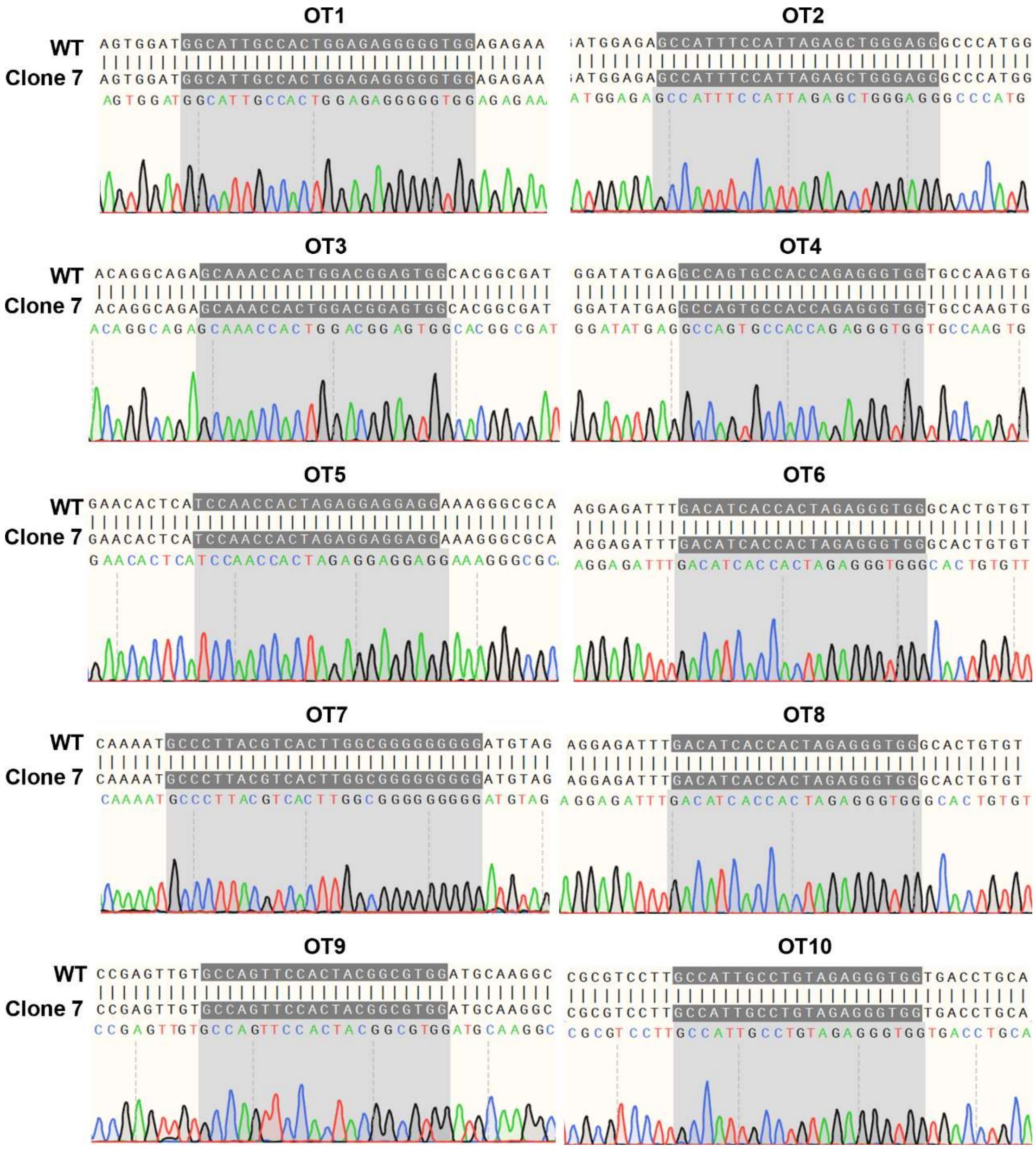

Figure S1. Analysis of potential CRISPR off-target sites. The 500~1000 bp sequences surrounding the top 10 potential off-target sites were amplified from the genomic DNA of FEV reporter cell line (H9-Clone#7), sanger sequencing of the sequences surrounding the off-target regions exposed no deviation of sequences in these regions of the edited clones compared from the parental untargeted cells. (The predicted off-target sites were highlighted in gray for sequencing chromatograms.)

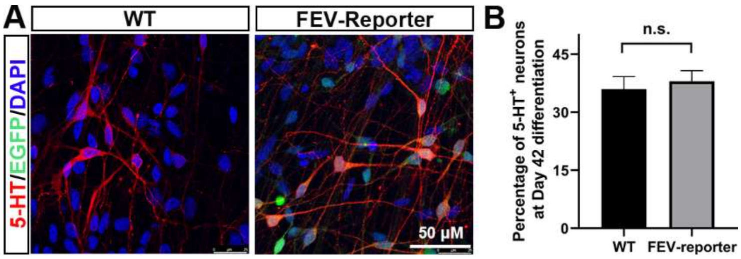

Figure S2. Comparison of the efficiency of serotonergic neuron differentiation between WT-H9 cell line and FEV-reporter cell line. (A) Representative images of immunofluorescence staining with 5-HT and EGFP in WT and engineered cells. Scale bar = 50 μm. **(B)** Quantification of 5-HT positive neurons derived from WT or engineered cells at day 42 of differentiation. Data are represented as mean ± SEM (n > 3 replicates, n.s.: not significant).

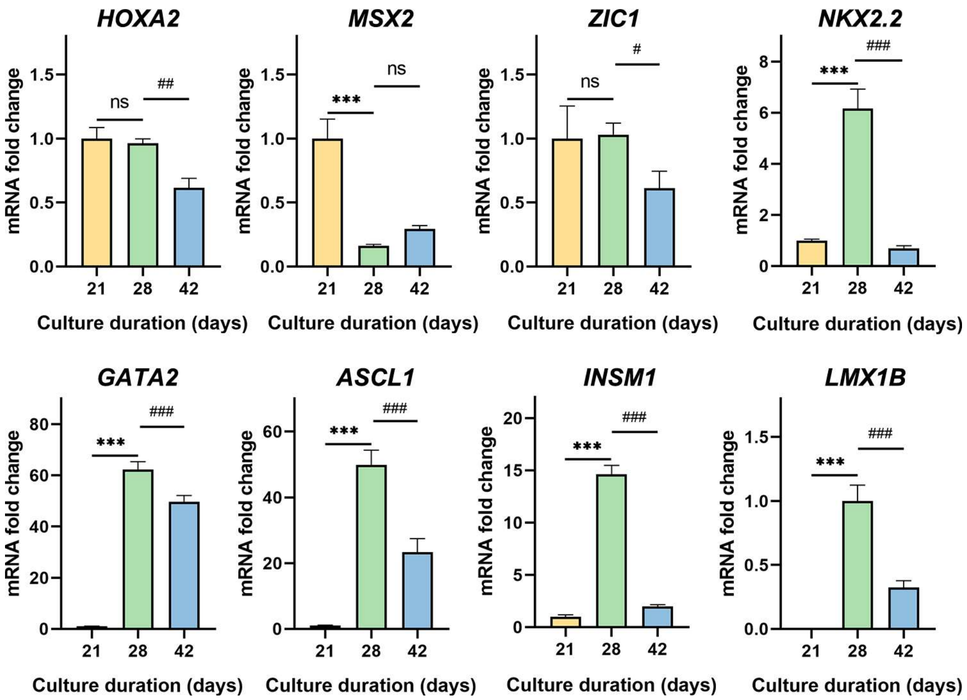

Figure S3. mRNA expression levels of hub genes (*HOXA2*, *MSX2*, *ZIC1*) and SN-associated genes (*NKX2.2*, *GATA2*, *ASCL1*, *INSM1*, *LMX1B*). (Day 28 vs day 21: * $p < 0.05$; ** $p < 0.01$; *** $p < 0.001$; ns, no significance. Day 42 vs day 28: # $p < 0.05$; ## $p < 0.01$; ### $p < 0.001$; ns, no significance.)

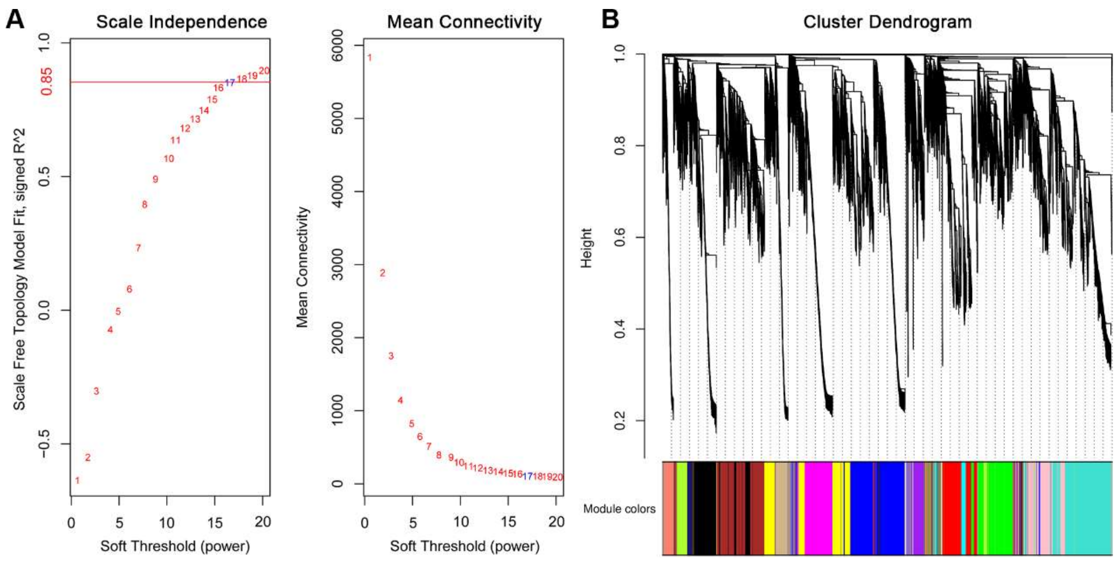

Figure S4. Establishment of the scale-free co-expression network and module detection in WGCNA. (A) Estimation of the soft thresholding power for a scale-free co-expression network and mean network connectivity under different soft thresholding power. The red line indicates a correlation coefficient of 0.85. **(B)** Clustering dendrograms of all DEGs, with dissimilarity based on topological overlap, together with assigned module colors. Altogether, 20 co-expression modules were constructed and displayed in different colors.

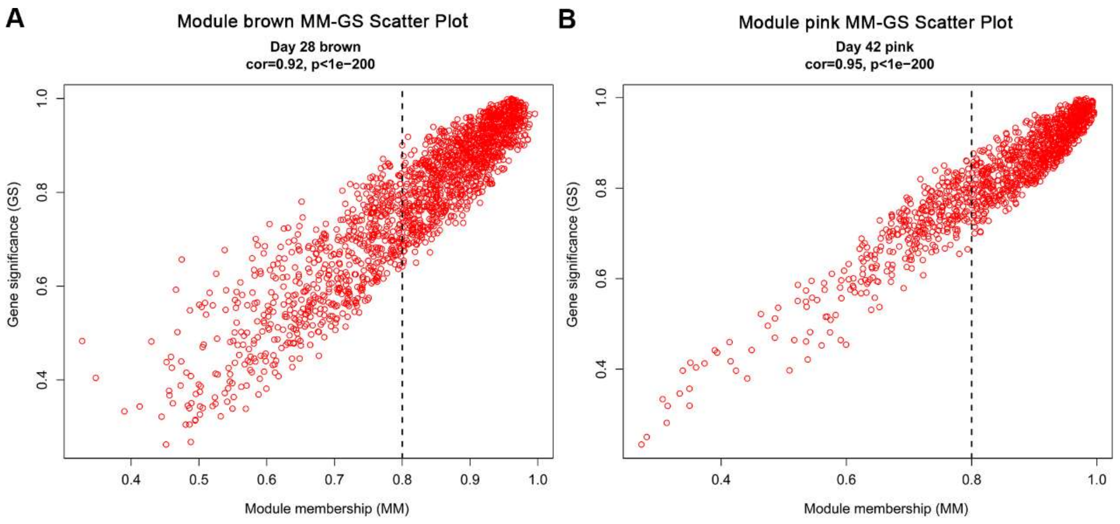

Figure S5. Scatterplots of gene significance (GS, y-axis) versus module membership (MM, x-axis) in module brown (A) and pink (B).

Table S1: List of reagents and software used in this study. Related to experimental procedures.

Reagent or Resource	Source	Identifier
**Antibodies**		
Goat polyclonal anti-SOX1	R&D system	Cat#AF3369; RRID: AB_2239879
Mouse monoclonal anti-NKX2.2	DSHB	Cat#74.5A5; RRID: AB_531794
Rabbit polyclonal anti-GATA2	Sigma	Cat#HPA005633; RRID: AB_1078954
Rabbit polyclonal anti-5-HT	ImmunoStar	Cat#20080; RRID: AB_572263
Goat polyclonal anti-5-HT	Abcam	Cat# ab66047; RRID: AB_1142794
Rabbit polyclonal anti-TPH2	Thermofisher	Cat#PA1-778; RRID: AB_2207687
Mouse monoclonal anti-Gata3	R&D systems	Cat#MAB6330; RRID: AB_10640512
Mouse monoclonal anti-GFP	Abcam	Cat#AB1218; RRID: AB_298911
Rabbit polyclonal anti-GFP	Beyotime	Cat#AG279; RRID: AB_2893351
Cy™3 AffiniPure Donkey Anti-Goat IgG (H+L)	Jackson ImmunoResearch	Cat#705-165-003; RRID:AB_2340411
Fluorescein (FITC) AffiniPure Donkey Anti-Goat IgG (H+L)	Jackson ImmunoResearch	Cat#705-095-003; RRID:AB_2340400
Fluorescein (FITC)-conjugated AffiniPure Donkey Anti-Mouse IgG (H+L)	Jackson ImmunoResearch	Cat#715-095-150; RRID:AB_2340792
Cy™3-conjugated AffiniPure Donkey Anti-Mouse IgG (H+L)	Jackson ImmunoResearch	Cat#715-165-15;; RRID:AB_2340813
Fluorescein (FITC)-conjugated AffiniPure Donkey Anti-Rabbit IgG (H+L)	Jackson ImmunoResearch	Cat#711-095-152; RRID:AB_2315776
Cy™3-conjugated AffiniPure Donkey Anti-Rabbit IgG (H+L)	Jackson ImmunoResearch	Cat#711-165-152; RRID:AB_2307443
**Chemicals, peptides, and recombinant proteins**		
SB 431542	TargetMol	Cat#T1726; CAS 301836-41-9
DMH1	TargetMol	Cat#T1942; CAS 1206711-16-1
CHIR 99021	TargetMol	Cat#T2310; CAS 252917-06-9

Purmorphamine	TargetMol	Cat#T1810;<br>CAS 483367-10-8
Y-27632 dihydrochloride	TargetMol	Cat#T1725;<br>CAS 129830-38-2
DAPT	TargetMol	Cat#T6202;<br>CAS 208255-80-5
Escitalopram Oxalate	TargetMol	Cat#T6493;<br>CAS 219861-08-2
FGF4	Novoprotein	Cat#CR08
GDNF	PeproTech	Cat#450-10
BDNF	PeproTech	Cat#450-02
TGFβ3	Novoprotein	Cat#CJ44
IGF1	PeproTech	Cat#100-11
bFGF	PeproTech	Cat#100-18B
poly-l-ornithine	Sigma-Ardrich	Cat#P3655
Vitamin C	Sigma-Ardrich	Cat#A4403
Matrigel	Corning	Cat#354277
mTeSR™1	Stemcell Technologies	Cat#85850
TrypLE	Gibco	Cat#12604021
DMEM/F12	Gibco	Cat#11330-032
Neurobasal	Gibco	Cat#21103049
N2	Gibco	Cat#17502048
B27	Gibco	Cat#12587010
Non-Essential amino acids (NEAA)	Gibco	Cat#11140050
GlutaMAX	Gibco	Cat#35050061
Knockout™ SR	Gibco	Cat#10828028
Laminin	Gibco	Cat#23017015
**Critical commercial assays**		
Serotonin high sensitive ELISA kit	IBL International GmbH	Cat#RE59141
Kapa genomic DNA extraction kit	Kapabiosystems	Cat#KK7102
**Experimental models: Cell lines**		
Human: H9 embryonic stem cells	WiCell Research Institute	WA01;<br>RRID: CVCL_9771
Human: FEV-EGFP stem cell line	This paper	N/A
**Recombinant DNA**		
PX330-sgRNA	N/A	N/A
FEV-T2A-EGFP	N/A	N/A
**Software and algorithms**		
GraphPad PRISM 6	GraphPad	http://www.graphpad.com;<br>RRID:SCR_002798
ImageJ	NIH	https://imagej.nih.gov/ij/;<br>RRID:SCR_003070

Table S2: List of oligo and primers.

Item	Orientation	Primer sequence (5’-3’)
**sgRNA oligo**		
Targeting FEV gene	GCCATTACCACTAGACGGGG	
**PCR primers**		
FEV-EGFP reporter: 5FR	forward	CCCCATCTCAGTCGTTACCC
	reverse	GTGCCCAGTCATAGCCGAATA
FEV-EGFP reporter: 3FR	forward	GCAACCTCCCCTTCTACGA
	reverse	GTTCCCCTTTTCACCCTCCT
FEV-EGFP reporter: FR	forward	AAAGCGCGGTAAGGCTACAC
	reverse	TCTCCCTGCTTTCCCCTAAC
**qPCR primers**		
NKX2.2	forward	CCTTCAGTACTCCCTGCAC
	reverse	TGTCATTGTCCGGTGACTC
ASCL1	forward	TTCACCAACTGGTTCTGAG
	reverse	TAAAGATGCAGGTTGTGCG
GATA2	forward	CAAGCTGCACAATGTTAACAG
	reverse	GACTTGTTGGACATCTTCCG
INSM1	forward	TCTACGAGTGCCATCACTG
	reverse	TACAAGGCCAGTAGGTCCT
LMX1B	forward	TTCCTGATGCGAGTCAACGAG
	reverse	GCAGTACAGTTTCCGATCCCG
HOXA2	forward	CGTCGCTCGCTGAGTGCCTG
	reverse	TGTCGAGTGTGAAAGCGTCGAGG
MSX2	forward	AATTCAGAAGATGGAGCGG
	reverse	TCATATGTCCTCCTACTCCTG
ZIC1	forward	CGCAAACACATGAAGGTCC
	reverse	GGAGGATTCGTAGCCAGAG
GAPDH	forward	TCAAGATCATCAGCAATGCC
	reverse	TTGATCTCTCCCTGATGTGTCT

Table S3: Off-target analysis of gene FEV-EGFP hPSC lines. Related to Figure S1.

Name	DNA sequence	Chromosome	Bugle type: size	Mismatches	Primer sequence (5'-3')	Indels
Off-target 1	GgCATTgCCACTGGAGAgGGGGT GG	chr8	DNA: 2	3	F: TCCCAGGTTGTATGCCTCTG<br>R: ACCTAGAAGAGGGGAGACACAA	ND
Off-target 2	GCCATTtCCAtTAGAGCtGGGAGG	chr15	RNA: 1	3	F: AGAAGCCCAGACGTTTTCCC<br>R: TCCCTGCTCCTTTGCTAACC	ND
Off-target 3	GCaA--ACCACTgGACGGaGTGG	chr8	RNA: 2	3	F: GTAGGCCCTGAAACGAGGTG<br>R: AAACCAGCCCCTCTTGTGTC	ND
Off-target 4	GCCAgTgCCACcAGA--GGGTGG	chr8	RNA: 2	3	F: ACAGGCAACCTACCCCTCT<br>R: GTGGGCAGCTAGAATTTTCCC	ND
Off-target 5	tCCA--ACCACTAGAgGaGGAGG	chr8	RNA: 2	3	F: CCTTCCAGGTGTTGCAGTCA<br>R: CGGGGTTCCACATCCTTTCT	ND
Off-target 6	GaCATcACCACTAGA-GGGtGGG	chr8	RNA: 1	3	F: TTGGAAGCCGTTCAGCACAA<br>R: CGGCACTTACCCACTGACAC	ND
Off-target 7	GCCcTTACGTCACTtGgCGGGGG GG	chr15	RNA: 2	3	F: TCCTGTCCATCTGTTTGGCG<br>R: CTCCTTACCTCTCCGACCGA	ND
Off-target 8	GaCATcACCACTAG--aGGGTGG	chr8	RNA: 2	3	F: TTGGAAGCCGTTCAGCACAA<br>R: CGGCACTTACCCACTGACAC	ND
Off-target 9	GCCAgTtCCACT--ACGGcGTGG	chr8	RNA: 2	3	F: TCTTGTTCTCTAATGCGTCGGA<br>R: GTCCGCAGAACAACAGGTACT	ND
Off-target 10	GCCATTgCCtgTAGA--GGGTGG	chr8	RNA: 2	3	F: AGGGGCTTTCAGGAGTCACA<br>R: GAGCATCGTGAGACCCATACA	ND
